# Copper bioreduction and nanoparticle synthesis by an enrichment culture from a former copper mine

**DOI:** 10.1111/1462-2920.16488

**Published:** 2023-09-11

**Authors:** Richard L. Kimber, Gretta Elizondo, Klaudia Jedyka, Christopher Boothman, Rongsheng Cai, Heath Bagshaw, Sarah J. Haigh, Victoria S. Coker, Jonathan R. Lloyd

**Affiliations:** ^1^ Department of Earth and Environmental Sciences, Williamson Research Centre for Molecular Environmental Science, School of Natural Sciences University of Manchester Manchester UK; ^2^ Department of Environmental Geosciences, Centre for Microbiology and Environmental Systems Science University of Vienna Vienna Austria; ^3^ Department of Materials University of Manchester Manchester UK; ^4^ SEM Shared Research Facility, School of Engineering University of Liverpool Liverpool UK

## Abstract

Microorganisms can facilitate the reduction of Cu^2+^, altering its speciation and mobility in environmental systems and producing Cu‐based nanoparticles with useful catalytic properties. However, only a few model organisms have been studied in relation to Cu^2+^ bioreduction and little work has been carried out on microbes from Cu‐contaminated environments. This study aimed to enrich for Cu‐resistant microbes from a Cu‐contaminated soil and explore their potential to facilitate Cu^2+^ reduction and biomineralisation from solution. We show that an enrichment grown in a Cu‐amended medium, dominated by species closely related to *Geothrix fermentans, Azospira restricta* and *Cellulomonas oligotrophica*, can reduce Cu^2+^ with subsequent precipitation of Cu nanoparticles. Characterisation of the nanoparticles with (scanning) transmission electron microscopy, energy‐dispersive x‐ray spectroscopy and electron energy loss spectroscopy supports the presence of both metallic Cu(0) and S‐rich Cu(I) nanoparticles. This study provides new insights into the diversity of microorganisms capable of facilitating copper reduction and highlights the potential for the formation of distinct nanoparticle phases resulting from bioreduction or biomineralisation reactions. The implications of these findings for the biogeochemical cycling of copper and the potential biotechnological synthesis of commercially useful copper nanoparticles are discussed.

## Graphical abstract

Microbial controls on copper redox transformations and nanoparticle synthesis are not well understood. In this study, a soil inoculum from a former copper mine was used to develop enrichment cultures grown in a Cu(II)‐amended medium. The enrichment culture, which became dominated by *Geothrix*, *Azospira*, and *Cellulomonas* species, was capable of reducing Cu2+ and precipitating Cu(0) and CuxS nanoparticles.

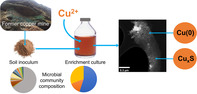

## INTRODUCTION

Microbial redox transformations of metals play a key role in the mobilisation or immobilisation of nutrients and contaminants in the environment (Gadd, 2010; Islam et al., 2004; Lloyd, 2003; Lovley, 1993; Unz & Shuttleworth, 1996). Copper (Cu) is an essential trace element for most forms of life, required as an important component in many enzymes and proteins. However, in excess it can also be highly toxic, inducing oxidative damage to cellular components and inhibiting cell function (Royer & Sharman, 2022). As such, Cu can be considered either a nutrient or contaminant depending on its concentration and bioavailability. In oxic soils, Cu is typically present as divalent Cu(II) and its mobility is limited by adsorption to soil organic matter (SOM) and mineral phases (Flogeac et al., 2004; Hering & Morel, 1988; Karlsson et al., 2006; Mclaren & Crawford, 1973; Parkman et al., 1999; Strawn et al., 2004; Weber et al., 2009). In reducing soils, microbial reduction of Cu(II) can result in the precipitation of metallic Cu(0) phases (Fulda et al., 2013; Hofacker et al., 2013; Mehlhorn et al., 2018; Weber et al., 2009). If subsequent microbial sulphate reduction occurs, transformation and replacement of metallic Cu(0) to S‐rich Cu_x_S upon reaction with biogenic sulphide has been reported (Fulda et al., 2013; Hofacker et al., 2013; Weber et al., 2009). While the precipitation of reduced Cu phases would typically be expected to limit Cu mobility (Mehlhorn et al., 2018), their mobilisation as cell‐associated colloids has been observed (Fulda et al., 2013; Hofacker et al., 2013; Weber et al., 2009). In addition to enhancing Cu mobilisation, the formation of colloidal Cu_x_S phases has also been shown to enhance the mobilisation of co‐contaminants such as Cd and Pb (Weber et al., 2009). Despite the importance of microbes in controlling Cu redox cycling and transport, as well as the potential mobilisation of other co‐contaminants, little is known about the organisms responsible for Cu reduction and mineralisation in the environment.

A *Clostridium* sp. was reported to facilitate the formation of Cu(0) nanoparticles (NPs) in a flooded soil, although formation of copper nanoparticles (CuNPs) in cell suspensions containing the *Clostridium* isolate was not directly observed (Hofacker et al., 2015). More recently, a member of the genus *Bacillus*, isolated from a copper mine in Brazil was shown to reduce aqueous Cu^2+^ to Cu^0^ (Gracioso et al., 2021). Our own recent work demonstrated that the model metal‐reducing bacterium, *Shewanella oneidensis*, was able to reduce Cu^2+^ and precipitate a mixture of intracellular and extracellular Cu(0) NPs (Kimber et al., 2018). Interestingly, we also observed biomineralisation of Cu_2_S‐like NPs in cell suspensions of another common metal‐reducing bacterium, *Geobacter sulfurreducens*, when challenged with Cu^2+^ (Kimber et al., 2020). *G. sulfurreducens* is incapable of respiring sulphate and so the formation of Cu_2_S NP by that organism suggests a pathway for Cu_x_S formation that does not require microbial sulphate reduction, in contrast to previous observations (Fulda et al., 2013; Hofacker et al., 2013; Weber et al., 2009). Despite this recent work, the diversity of microorganisms capable of Cu reduction is poorly understood, as are the potential microbial pathways for the formation of distinct biogenic products under anoxic conditions (e.g., metallic Cu(0) or sulphur‐rich CuNPs), limiting our understanding of the fate of Cu in the environment.

In addition to the environmental importance of microbial Cu reduction, biosynthesis of CuNPs offers a potential sustainable biotechnological route for the recovery of Cu from wastewaters, and the production of important Cu‐based catalytic NPs (Kimber et al., 2018). However, the toxicity of Cu towards microbial cells presents a potential barrier to this technology, limiting the production yield relative to more traditional physiochemical processes (Gawande et al., 2016; Kimber et al., 2018). Microbes from contaminated sites have been shown to display enhanced tolerance towards toxic metals and offer a promising source of novel microbial inocula capable of enhanced metal recovery and nanoparticle synthesis from toxic metals solutions (Das et al., 2014; Rostami et al., 2018; Wright et al., 2006).

In this study, we aimed to enrich and identify novel Cu^2+^‐reducing bacteria from a Cu‐contaminated soil at a former copper mine in the United Kingdom. Enrichment cultures were grown in a Cu‐amended medium to select for Cu‐tolerant bacteria that may display enhanced Cu^2+^ reduction and CuNP formation capabilities. The resulting biogenic CuNPs were characterised using transmission electron microscopy (TEM), scanning transmission electron microscopy (STEM), energy‐dispersive x‐ray spectroscopy (EDX), and electron energy loss spectroscopy (EELS). The composition of the enrichment cultures was also monitored by 16S rRNA gene sequencing to identify key organisms likely responsible for Cu^2+^ reduction.

## EXPERIMENTAL PROCEDURES

### Soil characterisation

To measure the pH of the soil samples (6.08 ± 0.13), we added 50 mL of deionised water to 5 g of soil taken from the study site. After mixing, the soil slurry was allowed to settle and the pH of the solution was measured. This was repeated for triplicate soil samples. Elemental composition of the study site soils was determined via microwave digestion and subsequent analysis via ICP‐AES (Perkin‐Elmer Optima 5300 dual view). Prior to the microwave digestion, the samples were dried at 75°C for 4 days. Once dried, each sample was ground into a fine powder to ensure homogeneity; 0.1 g of sample was added to TFM centrifuge tubes followed by 5 mL of 70% nitric acid. The tubes were capped with a PTFE stopper and a PFA screw cap before digestion in the microwave (MARS5 194AO7). The soil digestion was performed in triplicate in addition to two blanks to check for contamination. Mineral phase identification was carried out using powder x‐ray diffraction (XRD) crystallography on a Bruker D8 Advance with elemental imaging performed using a FEI XL30 ESEM‐FEG, equipped with the EDAX Gemini EDX system.

### Enrichment cultures

The enrichment medium comprised of LB broth (2.5 g/L), glycerol (50 mM) and fumarate (50 mM). The medium was prepared according to standard anoxic and aseptic techniques. Briefly, up to 1 L of the medium was prepared with deionised water; 30 mL of the medium was then decanted into 50 mL serum bottles, sealed and flushed with an 80:20 gas mix of N_2_:CO_2_ for 20 min to remove O_2_. The medium was then autoclaved and allowed to cool down. After cooling, a spike of filter sterilised anoxic Cu^2+^ solution, prepared using CuSO_4_, was added to give the desired concentration (300 or 500 μM). After autoclaving and Cu addition, the pH of the medium was 6.5 ± 0.1. The first enrichment was prepared via addition of 5 wt% of soil under anoxic conditions. After 4 weeks, fresh media were inoculated using a 5% v/v inoculum from the starting enrichment culture under anoxic and aseptic conditions. Each subsequent enrichment was prepared in the same way, with a 5% v/v inoculum from the previous enrichment. All cultures were incubated at 20°C. Growth was monitored via optical density (OD) measurements at 600 nm. Heat‐killed controls were prepared by autoclaving the medium immediately after inoculation. Cu‐free controls were by inoculating fresh media that contained no Cu^2+^ spike with an inoculum from a previous enrichment culture. Freshly prepared media with no added enrichment culture inocula were used as cell‐free controls.

### Microbial community analysis

16S rRNA gene sequencing was carried out on the soil used for the initial inoculum and on enrichments 5 and 7. DNA was extracted from 200 μL of sediment slurry or enrichment solution using a DNeasy PowerLyzer PowerSoil Kit (Qiagen, Manchester, UK), following the standard protocol supplied by the manufacturer. All extraction runs were performed with two negative extraction controls. Sequencing of PCR amplicons of 16S rRNA was conducted with the Illumina MiSeq platform (Illumina, San Diego, CA) targeting the V4 hyper variable region (forward primer, 515F, 5′‐GTGYCAGCMGCCGCGGTAA‐3′; reverse primer, 806R, 5′‐GGACTACHVGGGTWTCTAAT‐3′) for 2 × 250‐bp paired‐end sequencing (Illumina) (Kuippers et al., 2018). PCR amplification was performed using Roche FastStart High Fidelity PCR System (Roche Diagnostics Ltd, Burgess Hill, UK) in 50 μL reactions under the following conditions: initial denaturation at 95°C for 2 min, followed by 36 cycles of 95°C for 30 s, 55°C for 30 s, 72°C for 1 min and a final extension step of 5 min at 72°C. The PCR products were purified and normalised to ~20 ng each using the SequalPrep Normalization Kit (Fisher Scientific, Loughborough, UK). The PCR amplicons from all samples were pooled in equimolar ratios. The raw data obtained in this research were deposited to NCBI SRA (Sequence Read Archive; http://www.ncbi.nlm.nih.gov/sra/) under the project accession number: PRJNA976082.

### CuNP characterisation

Preparation of samples for TEM analysis has been described in detail previously (Kimber et al., 2018). In brief, 1 mL suspensions of the enrichment culture centrifuged at 14,900 rpm for 5 min, the supernatant discarded and the pellet resuspended in 1 mL deionised water. A sample (1.5 μL) of the washed cell suspension was pipetted onto a gold TEM grid with a holey‐carbon or carbon‐coated formvar support film and allowed to air dry in an anaerobic chamber. Samples were kept anaerobic until they were transferred into the TEM chamber, during which they would have been very briefly exposed to the atmosphere. TEM imaging and associated EDX analysis were performed in an JEOL 2100+ TEM fitted with a LaB6 Filament and operated at 200 kV. Images were taken with a Gatan Rio CCD camera and EDX collected with an Oxford X‐max 65 T EDS system and data analysed using Oxford AZtec software. STEM imaging, associated EDX analysis and EELS analysis of samples were performed in an aberration‐corrected Thermo Fisher Titan G2 STEM operated at 200 kV. High‐angle annular dark‐field (HAADF) STEM imaging was performed using a probe convergence angle of 21 mrad, a HAADF inner angle of 60 mrad and a probe current of ≈70 pA. EELS was performed using a Gatan Imaging Filter Quantum ER system with a 5 × 10^−3^ m entrance aperture and an energy dispersion of 1.2 eV. EDX analysis was performed using the Titan's Super‐X four silicon drift EDX detector system with a total collection solid angle of 0.7 srad.

## RESULTS

### Soil characterisation

Soil samples were collected from a topsoil adjacent to an outcrop exhibiting Cu mineralisation at a former mining site in the UK (53.29305"N and 2.20833"W). The pH of the soil samples was 6.08 ± 0.13. ICP analysis of soil digestions identified a range of heavy metal concentrations significantly above that of the normal background concentrations in the United Kingdom, as defined by the British Geological Survey (Johnson et al., 2012), with Cu concentrations of 2200 ± 54 mg/kg (Table 1). XRD analysis revealed a dominance of silicate minerals in the samples, including microcline, kaolinite, muscovite and quartz (Figure S1). In addition, the presence of two Cu mineral phases, native Cu and malachite (Cu₂CO₃(OH)₂), were also identified. Elemental imaging using environmental scanning electron microscopy (ESEM) and energy dispersive x‐ray spectroscopy (EDX) confirmed the presence of the heavy metals, Cu and Pb in the samples (Figure S2). The backscattered electron image revealed small (<20 μm) localised spots of electron dense elements present on the larger (>100 μm) grains. The relative dominance of Si and O in the larger grains, as revealed by elemental mapping, is consistent with the presence of silicate minerals identified by XRD. The smaller (<20 μm) electron‐dense spots correlated well to the locations of both Pb and S in the elemental maps. Cu appeared more uniformly distributed across the sample.

**TABLE 1:** Elemental composition of soil from the study site at Alderley Edge determined via ICP‐MS analysis following soil digestions (performed in triplicate with errors reported as the standard deviation) compared to the NBCs of selected heavy metals in English soils (Johnson et al., 2012).

Element	Concentration at study site (mg/kg)	Normal background concentrations (NBCs) in English soils (mg/kg) (Johnson et al., 2012)
Arsenic	649 ± 20	32
Calcium	3900 ± 2190	
Copper	2200 ± 54	62
Iron	13,600 ± 523	
Lead	9990 ± 230	180
Manganese	119 ± 8	
Potassium	5090 ± 325	
Sulphur	2250 ± 116	
Zinc	460 ± 17	

### Enrichment of Cu‐tolerant cultures

**FIGURE 1:**
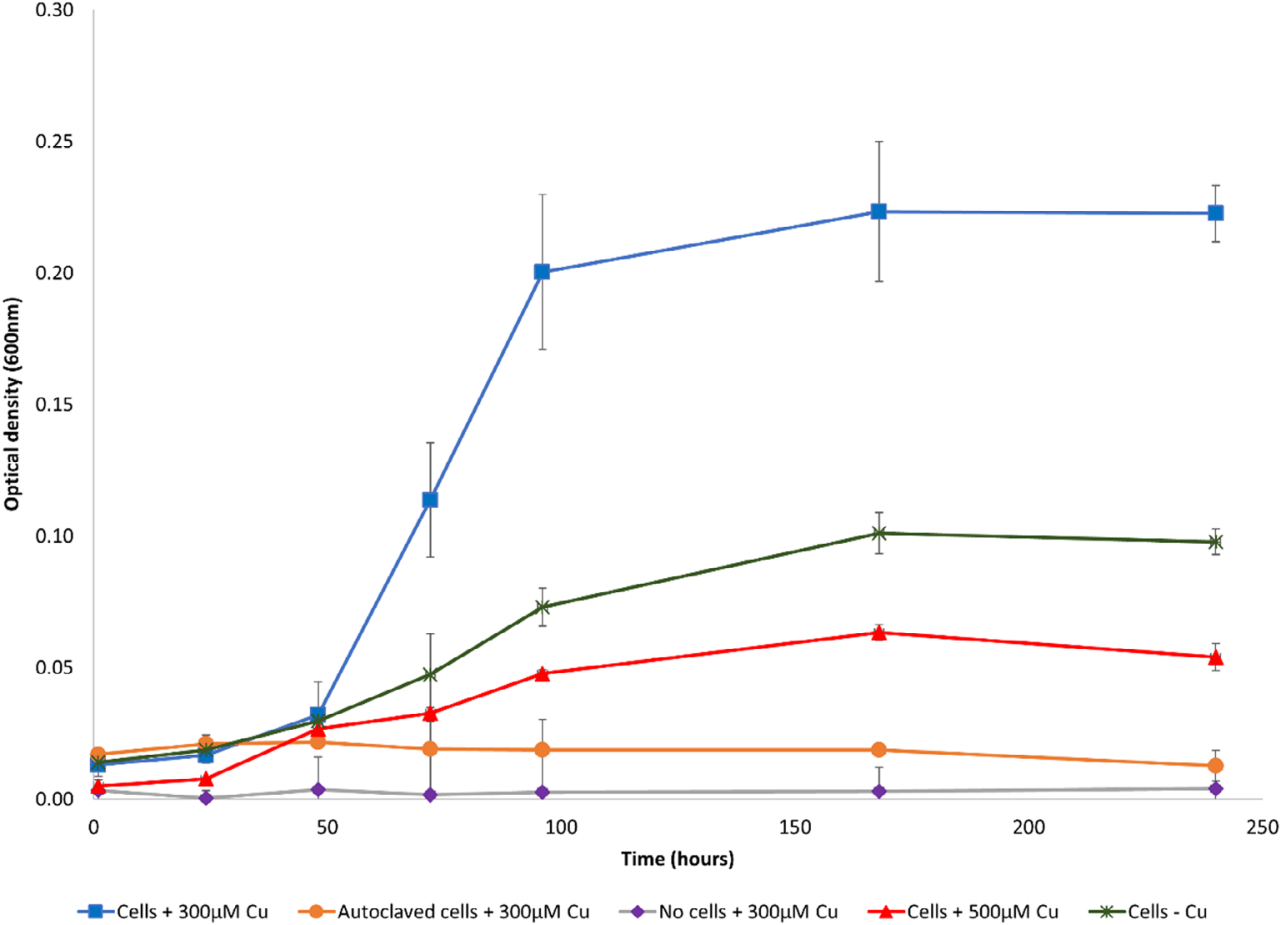
Representative growth curve of enrichment cultures as measured by OD_600_ over a 10‐day period. The data shown here display growth of cultures from Enrichment 7. All cultures, except for the no cell control (purple diamonds), which contained only the growth medium and Cu^2+^ spike, were inoculated with a 5% v/v inoculum from Enrichment 6. Blue squares and red triangles represent the enrichment cultures grown in the presence of a 300 or 500 μM Cu^2+^ spike, respectively. Orange circles represent growth from cultures that were autoclaved immediately after inoculation. Green crosses show growth of enrichment cultures grown in the absence of a Cu^2+^ spike. Error bars represent the standard deviation of triplicate enrichment cultures under each condition.

Initial attempts to establish enrichment cultures in defined media were unsuccessful (see Supporting Information). As such, a nutrient‐dense medium was selected. To establish enrichment cultures, we carried out a series of inoculations into an anoxic medium consisting of Lysogeny broth (LB) (2.5 g/L) supplemented with glycerol (50 mM) and fumarate (50 mM) as electron donors and acceptors, respectively. Cu^2+^ was added to the medium at an initial concentration of either 300 or 500 μM. The pH of the medium was 6.5 ± 0.1; 5 wt% soil from the sample site was used as the initial inoculum. After 7 days, the solution turned visibly cloudy (Figure S3). However, the high soil content prevented accurate determination of cell density via OD measurements. An enrichment series was then established using a 5% v/v inoculum of the initial enrichment into fresh medium. An active enrichment culture was maintained by inoculating fresh medium with a 5% v/v inoculum of the previous enrichment every 4 weeks. From the second inoculation in the enrichment series, the OD_600_ was measured (without background interference from the initial soil inoculum) to monitor microbial growth. The cultures were stable with similar growth yields observed over the 14 successive enrichment cultures that were produced. A representative growth curve from Enrichment 7 is shown in Figure 1. The highest OD was measured in inoculations using live cells in the presence of 300 μM Cu, reaching a maximum OD_600_ of 0.22 (±0.011) after 7 days. Inoculations of live cells in the presence of 500 μM Cu, resulted in a lower maximum OD_600_ of 0.06 after 7 days. The lower observed OD_600_ in the presence of higher Cu concentrations might be expected from increased toxicity resulting from a higher Cu concentration. Interestingly, in the absence of added Cu, inoculations with live cells only achieved a maximum OD_600_ of 0.10 after 7 days, lower than the OD observed in the presence of 300 μM Cu. The lower maximum OD_600_ observed in cultures grown in the absence of Cu relative to cells grown in Cu‐amended medium was consistent throughout the enrichment series; however, turbidity from Cu precipitation may have contributed to the higher OD_600_ in the Cu‐amended enrichments. No growth was observed in heat‐killed cell controls.

### Microbial community composition

**FIGURE 2:**
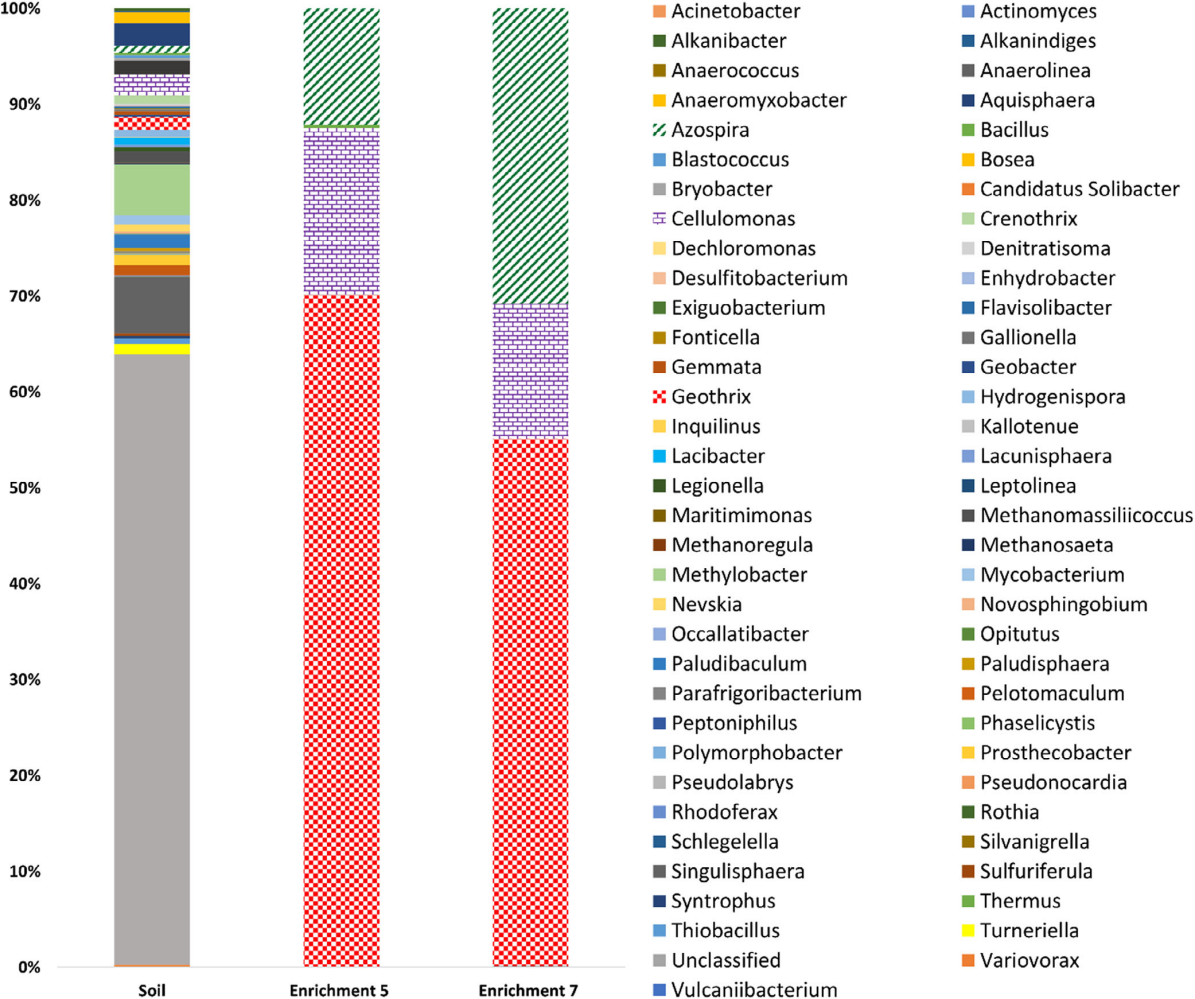
16S rRNA gene analysis of the microbial community of the sediment inoculum (left) and enrichment cultures grown in the Cu‐amended medium (middle and right). Results are presented at the genus level.

16S rRNA gene sequencing was used to analyse changes in the microbial community between the original soil and the Cu‐amended enrichment cultures (Figure 2). DNA extraction was carried out on Cultures 5 and 7 in the enrichment series. At the genus level, unclassified organisms made up the largest contribution to the initial soil inoculum at 63.7%. Species affiliated with the moderately acidophilic genus *Singulisphaera*, from the *Planctomycetaceae* family (Kulichevskaya et al., 2008), made the next largest contribution, with 5.9% of sequences. Species closely associated with the methanotrophic genus, *Methylobacter*, made up 5.3% of sequences. All other individual genera were represented by less than 2.5% of sequences. Enrichment cultures in the Cu^2+^‐amended medium produced a clear shift to a significantly less diverse microbial community, with 17 and 18 operational taxonomic units (OTUs) identified in Enrichments 5 and 7, respectively, compared to 249 OTUs identified in the initial soil inoculum. Sequences closely associated with the genera, *Cellulomonas*, *Geothrix* and *Azospira*, dominated both Enrichments 5 and 7, with all other genera combined making up <0.5% of sequences in each enrichment. Sequences affiliated with *Cellulomonas* made up 2.2% of the initial soil inoculum community but increased to 17.4% and 14.1% of the Enrichment 5 and 7 communities, respectively (>99.7% of these were most closely related to *Cellulomonas oligotrophica* strain Kc5 at 99% sequence similarity). This species is a facultative anaerobe, isolated previously during screening for dissimilatory Fe(III)‐reducing bacteria; however, its capacity for metal‐reduction is unknown (Hatayama et al., 2013). *Geothrix* species contributed 1.4% of sequences in the initial soil inoculum, increasing to 70% in Enrichment 5 and then decreasing to 55% in Enrichment 7. All sequences were affiliated with the *Geothrix* genus belonged to an OTU closely related to *Geothrix fermentans strain H5* (>99% sequence similarity). This species, a strict anaerobe, was isolated previously from a hydrocarbon‐contaminated aquifer and has been demonstrated to reduce Fe(III) and Mn(IV) (Coates et al., 1999). Sequences associated with the *Azospira* genus comprised 0.02% of the initial soil inoculum, increasing to 12.1% in Enrichment 5 and increasing again to 30.8% in Enrichment 7. In Enrichments 5 and 7, all sequences affiliated with the *Azospira* belonged to a single OTU most closely related to *Azospira restricta* strain SUA2 (98% sequence similarity), a nitrogen‐fixing bacterium isolated from groundwater (Bae et al., 2007; Mikes et al., 2021). The genera *Shewanella* and *Geobacter*, containing species observed to reduce Cu^2+^ based on our previous work (Kimber et al., 2018, 2020), were poorly represented here. No species affiliated with the *Shewanella* were detected, with species affiliated with the *Geobacter* comprising only 0.16% of sequences in the initial soil inoculum, and not detected in the enrichment cultures.

### Copper reduction and nanoparticle precipitation by enrichment cultures

**FIGURE 3:**
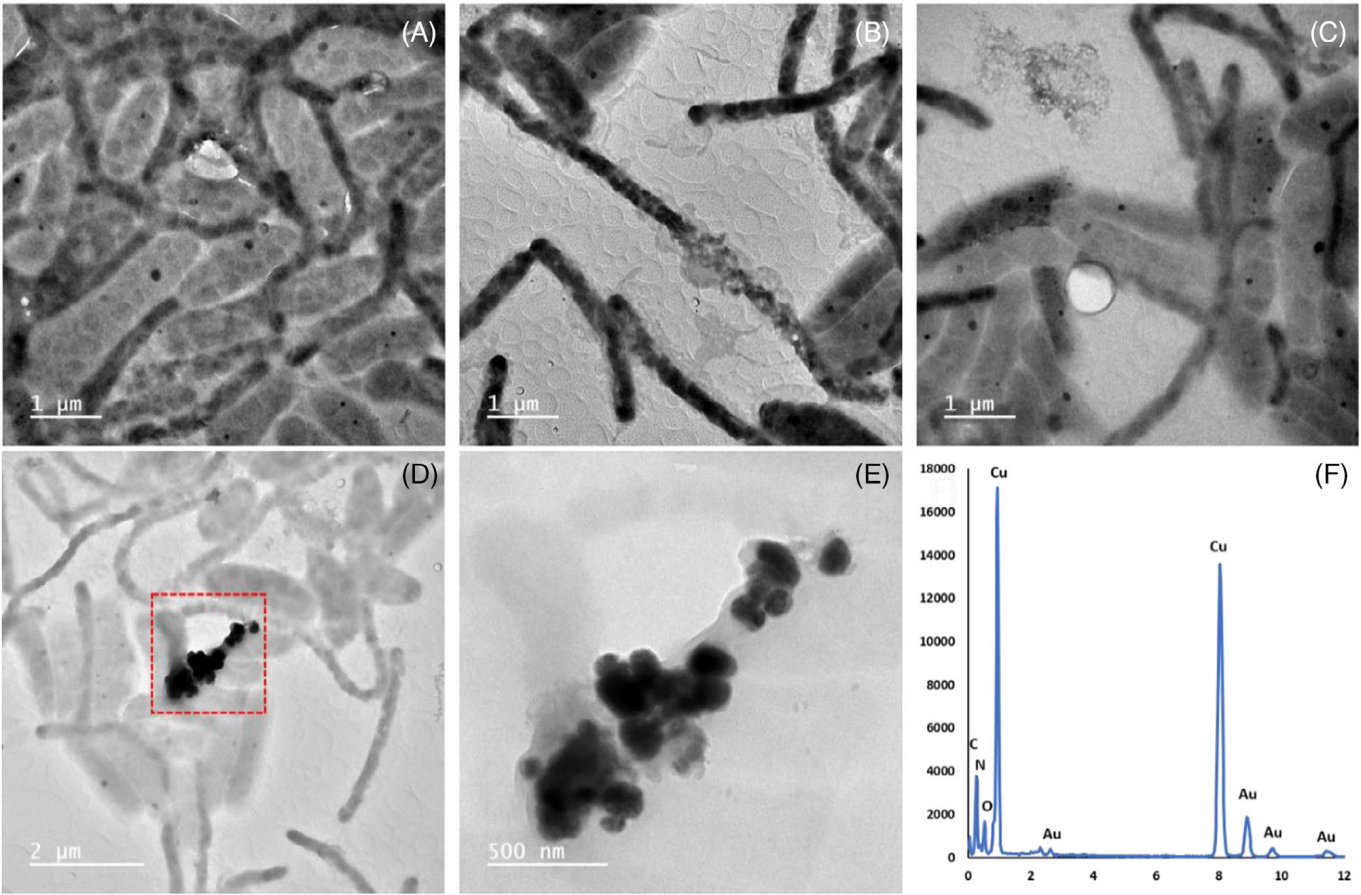
TEM images of cells and associated copper nanoparticles from an enrichment culture grown in Cu‐amended medium. Panel (E) is a magnified view of the red box in (D). The EDX spectra taken from the nanoparticles in (E) is shown in (F). The Au x‐ray signal in (F) is from the Au‐mesh of the holey carbon TEM support grid.

**FIGURE 4:**
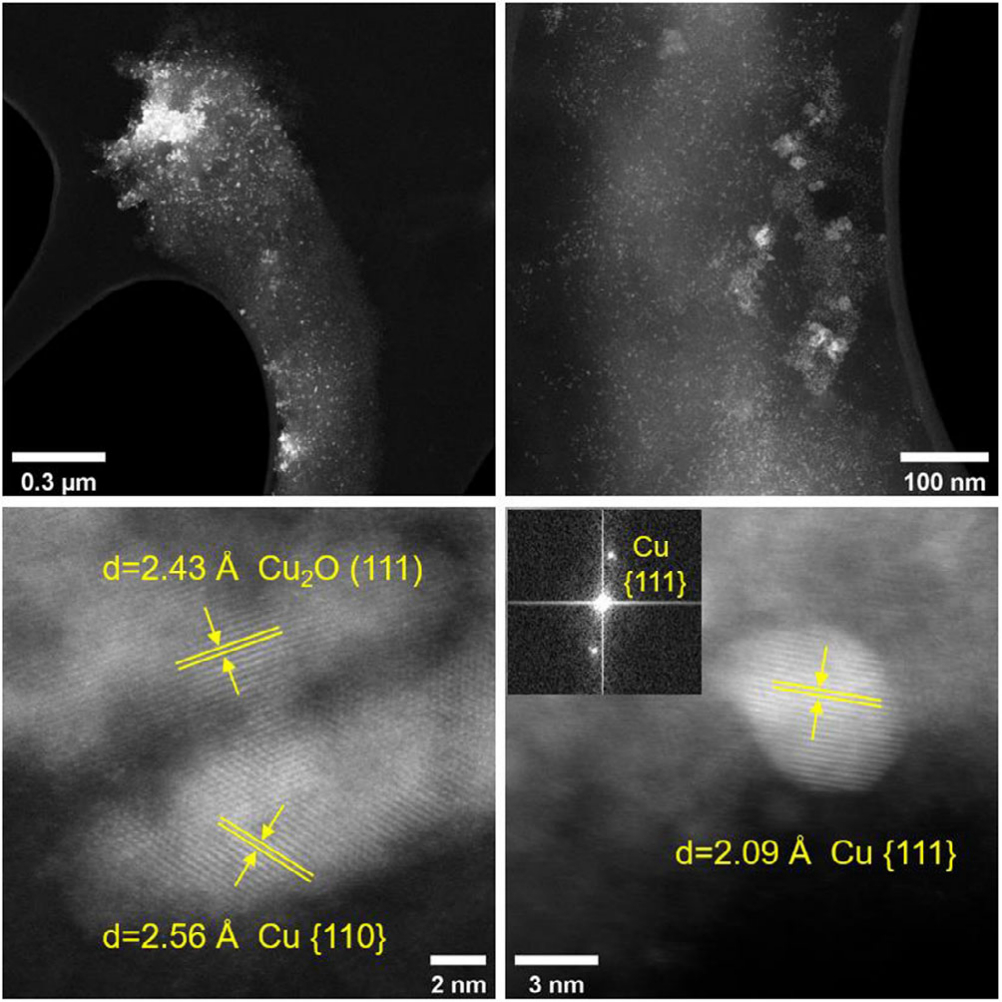
High‐angle annular dark‐field (HAADF) STEM images of enrichment culture cells coated in copper nanoparticles (top) and atomic resolution HAADF STEM images. The inset shows the corresponding Fast Fourier transforms (FFT) of the image.

To identify the potential for the enrichment cultures to reduce Cu^2+^ and to characterise any biomineralisation products, samples were analysed from Enrichment 7. These samples were taken after 7 days of incubation in the Cu^2+^‐amended medium, corresponding to the time taken to reach the highest biomass yields (estimated by OD; Figure 1). ICP‐AES confirmed that Cu removal from solution after 7 days incubation was greatest in the presence of live cells, with 44.5 ± 12.2% Cu removal from the initial starting concentration of 300 μM (Table S1). Heat‐killed (autoclaved) and no cell controls showed limited Cu removal after 7 days; 1.55 ± 3.23% and 7.67 ± 4.82%, respectively. To help characterise the mechanism of Cu removal from solution, a range of electron microscopy and spectroscopy analyses were performed on the samples taken after 7 days incubation. ESEM revealed the presence of significant electron‐dense particles associated with microbial cells (Figure S4A–C). The particles appeared to be present as discrete nanoparticles in the extracellular environment of the cells, as well as encrustations around cells. ESEM EDX point analysis clearly showed the discrete particles and encrustations were Cu‐rich (Figure S4D–F). TEM images with EDX analysis confirmed the presence of CuNP precipitates in the enrichment cultures (Figure 3). Two distinct rod‐like cell morphologies were observed in the TEM images: (i) cells approximately 2–3 μm in length and with a diameter of ~0.2 μm and (ii) cells of approximately 1–2 μm in length and with an approximate diameter of 0.5 μm. Copper nanoparticle precipitates were associated with both cell morphology types, occurring as individual nanoparticles and as agglomerates (Figure 3). Clusters of very small nanoparticles (<5 nm) were also found to be associated with cells (Figure 4). Cu‐free controls showed no evidence of CuNPs (Figure S5) suggesting limited carryover of CuNPs from previous enrichments. Cell‐free controls also showed no evidence of CuNP formation, ruling out abiotic precipitation of CuNPs (Figure S5).

### Characterisation of Cu nanoparticles

**FIGURE 5:**
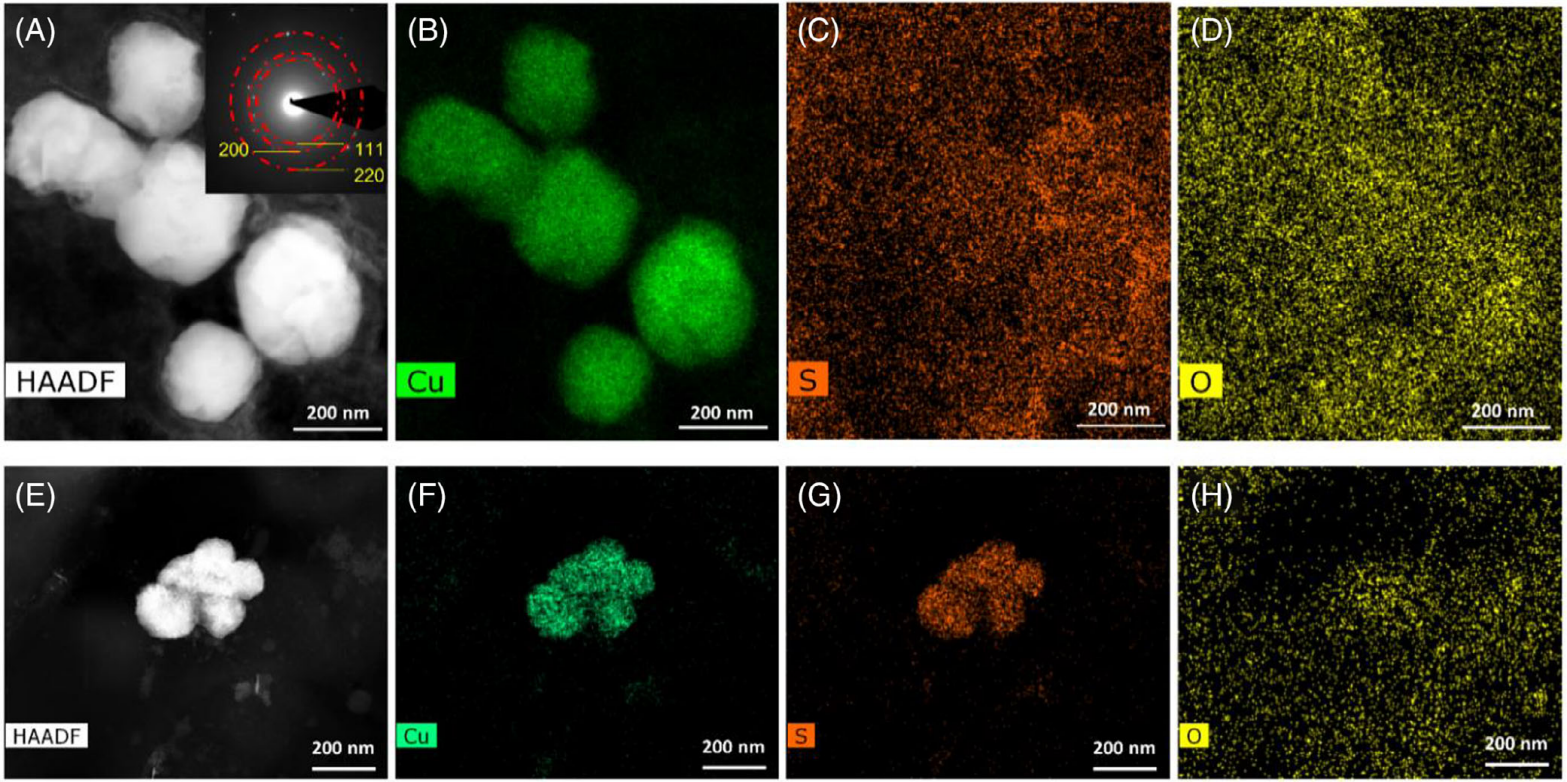
HAADF STEM images and corresponding EDX elemental maps of selected nanoparticles from the enrichment culture. The EDX elemental maps in (B–D) correspond to the nanoparticles in (A). EDX elemental maps in (F–H) correspond to the nanoparticles in (E). The inset shows selected area electron diffraction pattern taken from the particles in (A) with the pattern corresponding to face‐centred cubic Cu metal.

**FIGURE 6:**
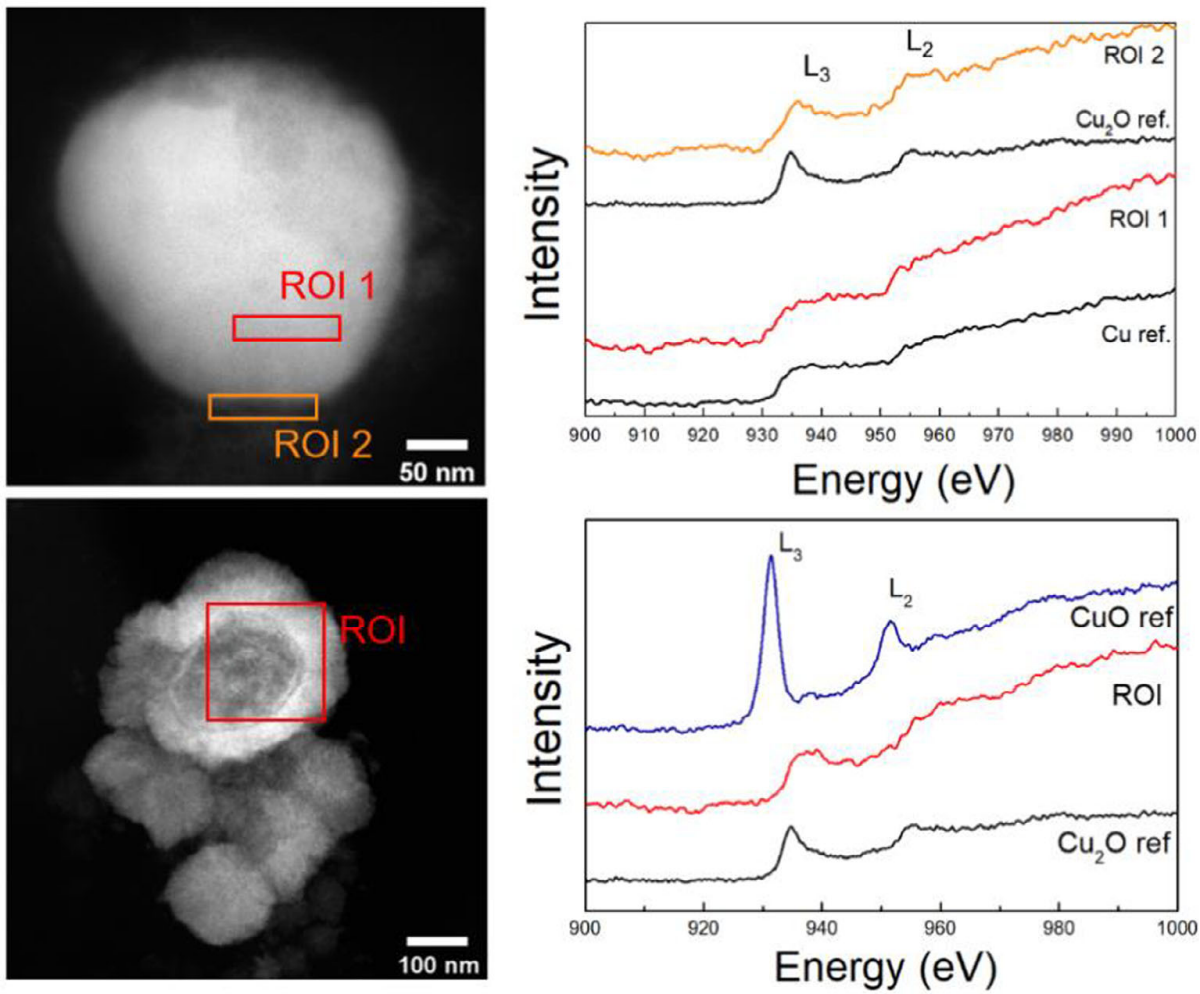
HAADF STEM images of selected nanoparticles (left) and corresponding EELS spectra (right) taken from the regions of interest (ROI) indicated.

The size of the Cu biominerals appears to be highly variable, as was observed in our previous work on Cu biomineralization by other anaerobic bacterial cultures (Kimber et al., 2018, 2020), ranging here from <5 nm to >200 nm in size (Figures 4, 5, 6). Selected area electron diffraction (SAED) patterns of larger (>200 nm) Cu biominerals were consistent with Cu(0) (Figure 5A). The interplanar lattice spacings and Fast Fourier Transforms (FFTs) from atomic resolution images of the smaller nanoparticles (<5 nm) were consistent with either Cu(0) or Cu_2_O (Figure 4). The presence of Cu_2_O is likely due to partial surface oxidation of the CuNPs as has been observed previously (Kimber et al., 2018). This is supported by EELS analysis that demonstrated that the bulk core of these biominerals is Cu(0) with Cu_2_O present as a thin surface layer (Figure 6). Taken together, these data clearly demonstrate that bioreduction of Cu^2+^ took place in the enrichment cultures with subsequent precipitation of Cu(0)‐NPs. In addition to the presence of Cu(0) biominerals, EDX elemental mapping performed in the STEM also identified sulphur‐rich Cu phases, ranging in size from <5 nm (Figure S6) to >50 nm (Figures 5E–H). A clear distinction between the Cu(0) biominerals with no S enrichment (Figures 5A–D) and the biominerals with Cu and S colocalisation (Figures 5E–H) can be seen from the STEM images, suggesting the presence of two distinct Cu‐rich phases. EELS data revealed the presence of a partially reduced Cu phase, with a spectrum similar to a Cu_2_O reference (Figure 6). However, we did not observe any significant correlation between Cu and O (Figure 5A–H). Furthermore, SAED patterns of these phases correspond to the expected Cu_2_S face centred cubic crystal structure (Figure S7). Taken together, the observation of partially reduced Cu(I), correlation between Cu and S, and SAED pattern supports the presence of Cu_2_S‐like phases. This would be consistent with our previous work where biomineralisation of Cu by *G. sulfurreducens* cells challenged with Cu^2+^ was attributed to Cu_2_S formation (Kimber et al., 2020). Precipitation of Cu_2_S NPs by the magnetotactic bacterium, *Desulfamplus magnetovallimortis*, has also recently been reported (Park et al., 2022).

## DISCUSSION

### Copper tolerance of enrichment cultures

In our previous work, the growth of Cu‐reducing bacteria, *G. sulfurreducens* and *S. oneidensis*, were shown to be strongly inhibited at aqueous Cu^2+^ concentrations of 100 μM and 10 μM, respectively (Kimber et al., 2018, 2020). In this study, growth of the enrichment culture was still observed at aqueous Cu concentrations up to 50‐fold higher (500 μM). Similarly, a Cu^2+^‐reducing bacterial strain from a copper mine in Brazil was isolated at aqueous Cu^2+^ concentrations of between 944 μM and 11.0 mM (Gracioso et al., 2021). However, whether these Cu‐reducing organisms enriched or isolated from Cu‐rich soils display enhanced Cu tolerance is difficult to ascertain due to the difference in media composition used across the different studies. For example, *S. oneidensis* and *G. sulfurreducens* were both grown in Cu^2+^‐amended fully defined media, whereas the enrichment culture here and the isolate from the Brazilian mine were both grown in a Cu^2+^‐amended complex medium containing LB broth, where the presence of tryptone and yeast extract may increase copper complexation and potentially decrease Cu toxicity (Davies et al., 1998; Sunda & Lewis, 1978). Interestingly, the growth of the enrichment cultures in this study as measured by OD, appeared to be enhanced in the presence of 300 μM Cu, relative to a no Cu control, potentially implying a beneficial role of Cu in their growth. However, OD measurements to determine the growth of enrichment cultures were made at a wavelength of 600 nm. Metallic CuNPs are also known to absorb light at similar wavelengths and hence, the increased absorbance values observed in the enrichment cultures grown in the presence of 300 μM Cu relative to no added Cu, may be partially explained by the precipitation of CuNPs in the former (Ismail et al., 2019; Pantidos et al., 2018; Ramanathan et al., 2013; Ramyadevi et al., 2011).

### Biosynthesis of CuNPs

The biomineralisation of both metallic and Cu_2_S‐like phases in our enrichment cultures is consistent with previous work investigating copper reduction by pure cultures (Gracioso et al., 2021; Kimber et al., 2018, 2020; Park et al., 2022). As discussed in our results, the enrichment cultures here were dominated by species most closely related to *C. oligotrophica*, *G. fermentans* and *A. restricta*. All other species combined made up less than 0.5% of the enrichment communities. As such, the observed Cu^2+^ bioreduction and biomineralisation of CuNPs is likely facilitated by one (or more) of the dominant organisms identified. None of the three dominant strains have previously been reported to reduce Cu^2+^ or to synthesise CuNPs. TEM images revealed the presence of Cu‐rich electron dense precipitates associated with two different cell morphologies, those with a diameter ~ 0.2 μm and those with a diameter ~ 0.5 μm. *G. fermentans* has been described as a rod‐shaped bacterium about 0.1 μm in diameter and ranging from 2 to 3 μm in length (Coates et al., 1999), closely resembling one of the cell morphologies observed in our TEM images (cells of length 2–3 μm and a diameter of ~0.2 μm). This size similarity, coupled with its known ability to reduce metals such as Fe(III) and Mn(IV), suggests *G. fermentans* may be one of the organisms involved in Cu^2+^ reduction and CuNP synthesis in these enrichment cultures. Both *C. oligotrophica* and *A. restricta* have been described as having a larger diameter than *G. fermentans* of 0.5–0.7 μm and 0.4–0.6 μm, respectively, and smaller cell lengths of between 1 and 3 μm (Bae et al., 2007; Hatayama et al., 2013). As such, the second cell morphology (cells of length 1–2 μm and a diameter of ~0.5 μm) seen in our TEM images and associated with CuNPs could plausibly comprise of either or both of these species. Although the metal‐reducing capabilities of *C. oligotrophica* and *A. restricta* are unknown, other species within the *Cellulomonas* genus have been shown to reduce metals, including Fe^3+^ and U^6+^ (Khanal et al., 2021; Sani et al., 2002).

Previous work with pure cultures has shown that the formation of distinct Cu biomineralisation products following copper bioreduction is dependent on the specific bacterial species involved (Gracioso et al., 2021; Kimber et al., 2018, 2020; Park et al., 2022). Although CuNPs were associated with two different cell morphologies in our TEM analyses, no clear association was observed between either cell morphology and a specific CuNPs phase (metallic or Cu_2_S‐like). As such, the relative role(s) and mechanism(s) of copper bioreduction and biomineralisation of the two distinct Cu phases by the enrichment culture species remains unclear. The formation of metallic CuNPs during copper bioreduction could plausibly result either via a single two‐electron transfer step, reducing Cu^2+^ directly to Cu^0^, or from a single‐electron transfer to Cu^2+^, resulting in the formation of transient Cu^+^ that could undergo a further one‐electron transfer or disproportionation to produce Cu^0^. The nutrient‐rich LB medium could provide a potential S source for the formation of the Cu_2_S‐like NPs; however, no CuNPs were observed in the abiotic control (Figure S5) and so a biotic mechanism for the precipitation of Cu_2_S‐like NPs appears necessary. Although we cannot completely rule out sulphate reduction in our enrichment cultures, the three organisms that dominated these enrichments (*G. fermentans*, *C. oligotrophica* and *A. restricta*) are not known to respire sulphate, suggesting an alternative source of reduced S is required. This is consistent with our previous study reporting the biomineralisation of similar Cu_2_S‐like NPs by *G. sulfurreducens*, a bacterium incapable of dissimilatory sulphate reduction. We speculate that assimilatory S metabolism could provide a sulphide source to precipitate a Cu_2_S‐like phase, either reacting with partially bioreduced Cu^+^ or by abiotically reducing Cu^2+^ to Cu^+^, followed by Cu_2_S precipitation. However, further work is required to elucidate the mechanisms of microbial copper reduction. Work is ongoing to attempt to isolate the individual strains from these enrichment cultures and identify the organism(s) capable of reducing Cu^2+^ (and compare their impact on Cu^2+^ using appropriate reference stains).

### Environmental and biotechnological significance

This study provides new insights into the potential diversity of microbial Cu reduction by demonstrating the bioreduction of Cu^2+^ and CuNP formation by an enrichment culture from a former copper mine, consisting of organisms not previously known to reduce Cu. The Cu biomineralisation products, a metallic Cu and an S‐rich Cu(I) phase, observed here are consistent with copper phases observed in anoxic soils, reported to result from microbial reduction of Cu(II) (Fulda et al., 2013; Hofacker et al., 2013; Mehlhorn et al., 2018; Weber et al., 2009). The biomineralisation of metallic CuNPs is associated with enhanced colloidal mobility of the Cu in soils under reducing conditions (Weber et al., 2009). Although the mechanism of microbial Cu(II) reduction in these soils is not understood, it is suggested that desorption of SOM‐bound Cu(II) is sustained by a Cu diffusion gradient driven by copper biomineralisation (Weber et al., 2009), providing a further source of aqueous or cell‐bound Cu^2+^ available for bioreduction. The results from the enrichment cultures in the current study, in agreement with previous studies using pure cultures (Gracioso et al., 2021; Kimber et al., 2018), suggest a potential pathway for the direct bioreduction of Cu^2+^ and formation of mobile metallic CuNPs colloids under soil reducing conditions, supporting previous field observations (Mehlhorn et al., 2018; Parkman et al., 1999; Strawn et al., 2004; Weber et al., 2009). This also has significance for the potential mobilisation of Cu from contaminated sites as this study demonstrates Cu reduction can be facilitated by organisms present in these heavy metal‐rich soils. This study also confirmed the biomineralisation of Cu_2_S‐like NPs. In soils, the formation of Cu_x_S phases was only observed following the onset of microbial sulphate‐reducing conditions, where biogenic sulphide was available to react with and transform the metallic CuNPs (Fulda et al., 2013; Hofacker et al., 2013; Weber et al., 2009). In sulphate‐depleted soils, only metallic CuNPs were observed suggesting microbial sulphate reduction was required for the formation of Cu_x_S phases in the soils studied (Fulda et al., 2013). However, previous work has shown that Cu_x_S NPs can form via direct transformation of Cu^2+^ in the absence microbial sulphate reduction (Kimber et al., 2020). The dominant organisms in the enrichment cultures in this study are also not known to respire sulphate, further supporting a pathway for the biomineralisation of Cu_x_S NPs in the absence of microbial sulphate reduction. As well as enhancing mobilisation of Cu as a colloidal phase in reducing soils, Cu_x_S NPs have also been shown to enhance the mobility of co‐contaminants (Weber et al., 2009). As such, this study has implications for the potential mobilisation of Cu and other co‐contaminants under a broader range of geochemical conditions than previously reported, for example, under more mildly reducing conditions.

In addition to the environmental importance, this study also has potential implications for the biotechnological synthesis of CuNPs. Our previous work demonstrated that metallic CuNPs produced by *S. oneidensis* could be applied successfully as catalysts in commercially important click‐chemistry reactions (Kimber et al., 2018). However, a limitation to this previous study was the relatively low yields of CuNPs produced due to the toxicity of Cu to the organism. The enrichment cultures developed in this study appear to display higher tolerance towards Cu^2+^ and a greater CuNP biomineralisation potential than in our previous studies on microbial CuNP synthesis (Kimber et al., 2018, 2020). As such, contaminated environments may prove a rich source of potential organisms for the enhanced synthesis of catalytically useful metal NPs. Furthermore, this study provides further evidence that the product of microbial copper reduction under anaerobic conditions is dependent upon the microbial species involved, presenting an opportunity for the biological synthesis of tailored metal NP catalysts (metallic Cu or Cu_x_S) for specific applications.

## AUTHOR CONTRIBUTIONS

**Richard L. Kimber:** Conceptualization (equal); formal analysis (equal); investigation (equal); methodology (equal); writing – original draft (equal). **Gretta Elizondo:** Investigation (supporting); methodology (supporting). **Klaudia Jedyka:** Investigation (supporting); methodology (supporting). **Christopher Boothman:** Data curation (supporting); formal analysis (supporting); investigation (supporting). **Rongsheng Cai:** Investigation (supporting); methodology (supporting); writing – original draft (supporting); writing – review and editing (supporting). **Heath Bagshaw:** Investigation (supporting); methodology (supporting); writing – review and editing (supporting). **Sarah J. Haigh:** Investigation (supporting); methodology (supporting); writing – original draft (supporting); writing – review and editing (supporting). **Victoria S. Coker:** Conceptualization (equal); methodology (supporting); writing – review and editing (supporting). **Jonathan R. Lloyd:** Conceptualization (equal); funding acquisition (lead); investigation (supporting); methodology (equal); project administration (lead); writing – review and editing (supporting).

## CONFLICT OF INTEREST STATEMENT

The authors declare no conflicts of interest.

## Supporting information


**Data S1:** Supporting Information.

## Data Availability

The data that support the findings of this study are available from the corresponding author upon reasonable request.

## References

[emi16488-bib-0001] Bae, H.S. , Rash, B.A. , Rainey, F.A. , Nobre, M.F. , Tiago, I. , da Costa, M.S. et al. (2007) Description of *Azospira restricta* sp. nov., a nitrogen‐fixing bacterium isolated from groundwater. International Journal of Systematic and Evolutionary Microbiology, 57, 1521–1526. Available from: 10.1099/ijs.0.64965-0 17625187

[emi16488-bib-0002] Coates, J.D. , Ellis, D.J. , Gaw, C.V. & Lovley, D.R. (1999) *Geothrix fermentans* gen. nov., sp. nov., a novel Fe(III)‐reducing bacterium from a hydrocarbon‐contaminated aquifer. International Journal of Systematic Bacteriology, 49(Pt 4), 1615–1622. Available from: 10.1099/00207713-49-4-1615 10555343

[emi16488-bib-0003] Das, V.L. , Thomas, R. , Varghese, R.T. , Soniya, E.V. , Mathew, J. & Radhakrishnan, E.K. (2014) Extracellular synthesis of silver nanoparticles by the *Bacillus* strain CS 11 isolated from industrialized area. 3 Biotech, 4, 121–126. Available from: 10.1007/s13205-013-0130-8 PMC3964251 28324441

[emi16488-bib-0004] Davies, C.M. , Apte, S.C. & Johnstone, A.L. (1998) A bacterial bioassay for the assessment of copper bioavailability in freshwaters. Environmental Toxicology and Water Quality, 13, 263–271. Available from: 10.1002/(SICI)1098-2256

[emi16488-bib-0005] Flogeac, K. , Guillon, E. & Aplincourt, M. (2004) Surface complexation of copper(II) on soil particles: EPR and XAFS studies. Environmental Science & Technology, 38, 3098–3103. Available from: 10.1021/es049973f 15224741

[emi16488-bib-0006] Fulda, B. , Voegelin, A. , Ehlert, K. & Kretzschmar, R. (2013) Redox transformation, solid phase speciation and solution dynamics of copper during soil reduction and reoxidation as affected by sulfate availability. Geochimica et Cosmochimica Acta, 123, 385–402. Available from: 10.1016/j.gca.2013.07.017

[emi16488-bib-0007] Gadd, G.M. (2010) Metals, minerals and microbes: geomicrobiology and bioremediation. Microbiology (Reading), 156, 609–643. Available from: 10.1099/mic.0.037143-0 20019082

[emi16488-bib-0008] Gawande, M.B. , Goswami, A. , Felpin, F.‐X. , Asefa, T. , Huang, X. , Silva, R. et al. (2016) Cu and Cu‐based nanoparticles: synthesis and applications in catalysis. Chemical Reviews, 116, 3722–3811. Available from: 10.1021/acs.chemrev.5b00482 26935812

[emi16488-bib-0009] Gracioso, L.H. , Peña‐Bahamonde, J. , Karolski, B. , Borrego, B.B. , Perpetuo, E.A. , do Nascimento, C.A.O. et al. (2021) Copper mining bacteria: converting toxic copper ions into a stable single‐atom copper. Science Advances, 7, eabd9210. Available from: 10.1126/sciadv.abd9210 33893098 PMC8064636

[emi16488-bib-0010] Hatayama, K. , Esaki, K. & Ide, T. (2013) *Cellulomonas soli* sp. nov. and *Cellulomonas oligotrophica* sp. nov., isolated from soil. International Journal of Systematic and Evolutionary Microbiology, 63, 60–65. Available from: 10.1099/ijs.0.038364-0 22328604

[emi16488-bib-0011] Hering, J.G. & Morel, F.M.M. (1988) Humic acid complexation of calcium and copper. Environmental Science & Technology, 22, 1234–1237. Available from: 10.1021/es00175a018 22148621

[emi16488-bib-0012] Hofacker, A.F. , Behrens, S. , Voegelin, A. , Kaegi, R. , Lösekann‐Behrens, T. , Kappler, A. et al. (2015) *Clostridium* species as metallic copper‐forming bacteria in soil under reducing conditions. Geomicrobiology Journal, 32, 130–139. Available from: 10.1080/01490451.2014.933287

[emi16488-bib-0013] Hofacker, A.F. , Voegelin, A. , Kaegi, R. , Weber, F.‐A. & Kretzschmar, R. (2013) Temperature‐dependent formation of metallic copper and metal sulfide nanoparticles during flooding of a contaminated soil. Geochimica et Cosmochimica Acta, 103, 316–332. Available from: 10.1016/j.gca.2012.10.053

[emi16488-bib-0014] Islam, F.S. , Gault, A.G. , Boothman, C. , Polya, D.A. , Charnock, J.M. , Chatterjee, D. et al. (2004) Role of metal‐reducing bacteria in arsenic release from Bengal delta sediments. Nature, 430, 68–71. Available from: 10.1038/nature02638 15229598

[emi16488-bib-0015] Ismail, M. , Gul, S. , Khan, M.I. , Khan, M.A. , Asiri, A.M. & Khan, S.B. (2019) Green synthesis of zerovalent copper nanoparticles for efficient reduction of toxic azo dyes congo red and methyl orange. Green Processing and Synthesis, 8, 135–143. Available from: 10.1515/gps-2018-0038

[emi16488-bib-0016] Johnson, C.C. , Ander, E.L. , Cave, M.R. & Palumbo‐Roe, B. (2012) Normal background concentrations (NBCs) of contaminants in English soils: final project report. Nottingham, UK: British Geological Survey.

[emi16488-bib-0017] Karlsson, T. , Persson, P. & Skyllberg, U. (2006) Complexation of copper(ll) in organic soils and in dissolved organic matter—EXAFS evidence for chelate ring structures. Environmental Science & Technology, 40, 2623–2628. Available from: 10.1021/es052211f 16683601

[emi16488-bib-0018] Khanal, A. , Hur, H.‐G. , Fredrickson, J.K. & Lee, J.‐H. (2021) Direct and indirect reduction of Cr(VI) by fermentative Fe(III)‐reducing cellulomonas sp. strain cellu‐2a. Journal of Microbiology and Biotechnology, 31, 1519–1525. Available from: 10.4014/jmb.2107.07038 34489371 PMC9706010

[emi16488-bib-0019] Kimber, R.L. , Bagshaw, H. , Smith, K. , Buchanan, D.M. , Coker, V.S. , Cavet, J.S. et al. (2020) Biomineralization of Cu(2)S nanoparticles by *Geobacter sulfurreducens* . Applied and Environmental Microbiology, 86, e00967‐20. Available from: 10.1128/AEM.00967-20 32680873 PMC7480366

[emi16488-bib-0020] Kimber, R.L. , Lewis, E.A. , Parmeggiani, F. , Smith, K. , Bagshaw, H. , Starborg, T. et al. (2018) Biosynthesis and characterization of copper nanoparticles using *Shewanella oneidensis*: application for click chemistry. Small, 14, 1703145. Available from: 10.1002/smll.201703145 29359400

[emi16488-bib-0021] Kuippers, G. , Boothman, C. , Bagshaw, H. , Ward, M. , Beard, R. , Bryan, N. et al. (2018) The biogeochemical fate of nickel during microbial ISA degradation; implications for nuclear waste disposal. Scientific Reports, 8, 8753. Available from: 10.1038/s41598-018-26963-8 29884890 PMC5993814

[emi16488-bib-0022] Kulichevskaya, I.S. , Ivanova, A.O. , Baulina, O.I. , Bodelier, P.L.E. , Damsté, J.S.S. & Dedysh, S.N. (2008) *Singulisphaera acidiphila* gen. nov., sp. nov., a non‐filamentous, *Isosphaera*‐like planctomycete from acidic northern wetlands. International Journal of Systematic and Evolutionary Microbiology, 58, 1186–1193. Available from: 10.1099/ijs.0.65593-0 18450711

[emi16488-bib-0023] Lloyd, J.R. (2003) Microbial reduction of metals and radionuclides. FEMS Microbiology Reviews, 27, 411–425. Available from: 10.1016/S0168-6445(03)00044-5 12829277

[emi16488-bib-0024] Lovley, D.R. (1993) Dissimilatory metal reduction. Annual Review of Microbiology, 47, 263–290. Available from: 10.1146/annurev.mi.47.100193.001403 8257100

[emi16488-bib-0025] Mclaren, R.G. & Crawford, D.V. (1973) Studies on soil copper. Journal of Soil Science, 24, 443–452. Available from: 10.1111/j.1365-2389.1973.tb02311.x

[emi16488-bib-0026] Mehlhorn, J. , Besold, J. , Lezama Pacheco, J.S. , Gustafsson, J.P. , Kretzschmar, R. & Planer‐Friedrich, B. (2018) Copper mobilization and immobilization along an organic matter and redox gradient—insights from a mofette site. Environmental Science & Technology, 52, 13698–13707. Available from: 10.1021/acs.est.8b02668 30199245

[emi16488-bib-0027] Mikes, M.C. , Moe, W.M. & Hotopp, J.C.D. (2021) Genome sequence of the type strain *Azospira restricta* SUA2 (DSM 18626). Microbiology Resource Announcements, 10, e00156‐21. Available from: 10.1128/MRA.00156-21 33958413 PMC8103858

[emi16488-bib-0028] Pantidos, N. , Edmundson, M.C. & Horsfall, L. (2018) Room temperature bioproduction, isolation and anti‐microbial properties of stable elemental copper nanoparticles. New Biotechnology, 40, 275–281. Available from: 10.1016/j.nbt.2017.10.002 29017818 PMC5734607

[emi16488-bib-0029] Park, Y. , Eyal, Z. , Pekker, P. , Chevrier, D.M. , Lefèvre, C.T. , Arnoux, P. et al. (2022) Periplasmic bacterial biomineralization of copper sulfide nanoparticles. Advanced Science, 9, 2203444. Available from: 10.1002/advs.202203444 35975419 PMC9534983

[emi16488-bib-0030] Parkman, R.H. , Charnock, J.M. , Bryan, N.D. , Livens, F.R. & Vaughan, D.J. (1999) Reactions of copper and cadmium ions in aqueous solution with goethite, lepidocrocite, mackinawite, and pyrite. American Mineralogist, 84, 407–419. Available from: 10.2138/am-1999-0326

[emi16488-bib-0031] Ramanathan, R. , Field, M.R. , O'Mullane, A.P. , Smooker, P.M. , Bhargava, S.K. & Bansal, V. (2013) Aqueous phase synthesis of copper nanoparticles: a link between heavy metal resistance and nanoparticle synthesis ability in bacterial systems. Nanoscale, 5, 2300–2306. Available from: 10.1039/C2NR32887A 23223802

[emi16488-bib-0032] Ramyadevi, J. , Jeyasubramanian, K. , Marikani, A. , Rajakumar, G. , Rahuman, A.A. , Santhoshkumar, T. et al. (2011) Copper nanoparticles synthesized by polyol process used to control hematophagous parasites. Parasitology Research, 109, 1403–1415. Available from: 10.1007/s00436-011-2387-3 21526405

[emi16488-bib-0033] Rostami, H. , Khosravi, F. , Mohseni, M. & Rostami, A.A. (2018) Biosynthesis of Ag nanoparticles using isolated bacteria from contaminated sites and its application as an efficient catalyst for hydrazine electrooxidation. International Journal of Biological Macromolecules, 107, 343–348. Available from: 10.1016/j.ijbiomac.2017.08.179 28870750

[emi16488-bib-0034] Royer, A. & Sharman, T. (2022) StatPearls. Treasure Island, FL: StatPearls Publishing.

[emi16488-bib-0035] Sani, R.K. , Peyton, B.M. , Smith, W.A. , Apel, W.A. & Petersen, J.N. (2002) Dissimilatory reduction of Cr(VI), Fe(III), and U(VI) by *Cellulomonas* isolates. Applied Microbiology and Biotechnology, 60, 192–199. Available from: 10.1007/s00253-002-1069-6 12382063

[emi16488-bib-0036] Strawn, D.G. , Palmer, N.E. , Furnare, L.J. , Goodell, C. , Amonette, J.E. & Kukkadapu, R.K. (2004) Copper sorption mechanisms on smectites. Clays and Clay Minerals, 52, 321–333. Available from: 10.1346/CCMN.2004.0520307

[emi16488-bib-0037] Sunda, W.G. & Lewis, J.A.M. (1978) Effect of complexation by natural organic ligands on the toxicity of copper to a unicellular alga, *Monochrysis lutheri* . Limnology and Oceanography, 23, 870–876. Available from: 10.4319/lo.1978.23.5.0870

[emi16488-bib-0038] Unz, R.F. & Shuttleworth, K.L. (1996) Microbial mobilization and immobilization of heavy metals. Current Opinion in Biotechnology, 7, 307–310. Available from: 10.1016/S0958-1669(96)80035-8 8785435

[emi16488-bib-0039] Weber, F.‐A. , Voegelin, A. , Kaegi, R. & Kretzschmar, R. (2009) Contaminant mobilization by metallic copper and metal sulphide colloids in flooded soil. Nature Geoscience, 2, 267–271. Available from: 10.1038/ngeo476

[emi16488-bib-0040] Wright, M.S. , Peltier, G.L. , Stepanauskas, R. & McArthur, J.V. (2006) Bacterial tolerances to metals and antibiotics in metal‐contaminated and reference streams. FEMS Microbiology Ecology, 58, 293–302. Available from: 10.1111/j.1574-6941.2006.00154.x 17064270

