# Supplementary material for: Copper bioreduction and nanoparticle synthesis by an enrichment culture from a former copper mine
Source: Environ Microbiol. 2023 Sep 11;25(12):3139–50. doi: 10.1111/1462-2920.16488 (PMC10946571; doi:10.1111/1462-2920.16488)
Supplement: Supplementary file 1 — Data S1: Supporting Information. [file EMI-25-3139-s001.docx]

**Copper bioreduction and nanoparticle synthesis by an enrichment culture from a former copper mine**

**Supplementary Information**

 Kimber^1,2*^, G. Elizondo^1^, K. Jedyka^1^, C. Boothman^1^, R. Cai^3^, H. Bagshaw^4^, S.J. Haigh^3^, V.S. Coker^1^, J.R. Lloyd^1^

^1^*Department of Earth and Environmental Sciences, Williamson Research Centre for Molecular Environmental Science, University of Manchester, UK*

^2^*Department of Environmental Geosciences, Centre for Microbiology and Environmental Systems Science, University of Vienna, Austria*

^3^*Department of Materials, University of Manchester, UK*

^4^*SEM Shared Research Facility, School of Engineering, University of Liverpool, U.K*

^*^*Email:* [*Richard.kimber@univie.ac.at*](mailto:Richard.kimber@univie.ac.at)

**SI text**

At first, we attempted to establish enrichment cultures in anoxic minimal media in the presence and absence of additional Cu^2+^. In these initial experiments, 5-10 wt% of soil was added to defined medium which contained either, acetate (50mM), glucose (50mM), or lactate (50mM) as the electron donor and fumarate (50mM) as electron acceptor ^1^. The media were supplemented with either 0 μM, 10 μM, 100 μM, or 1 mM additional Cu^2+^. Once inoculated, the cultures were incubated at 20 or 30 °C. There was no observable growth in any of these initial inoculations. Subsequent inoculations were prepared using 10% v/v of the initial inoculation into fresh media. Again, no observable growth was seen and no increase in optical density (OD) was observed.

**References**

1. H. Muhamadali, Y. Xu, D. I. Ellis, J. W. Allwood, N. J. W. Rattray, E. Correa, H. Alrabiah, J. R. Lloyd and R. Goodacre, *Applied and Environmental Microbiology*, 2015, **81**, 3288-3298. DOI:10.1128/AEM.00294-15

|  | Live cells | Autoclaved cells | No Cells |
| --- | --- | --- | --- |
| Initial concentration (μM) | 319 (±48.2) | 330 (±5.78) | 320 (±19.5) |
| Concentration after 7 days (μM) | 183 (±76.9) | 325 (±16.4) | 296 (±33.4) |
| Percentage removal from solution (%) | 44.5 (12.2) | 1.55 (±3.23) | 7.67 (±4.82) |

**Table S1.** ICP-AES analysis of Cu in solution over the first 7-day growth period of the enrichment cultures and heat-killed (autoclaved) and cell free controls. Samples for characterization of CuNPs were taken on day 7. Errors represent the standard deviation from cultures and controls performed in triplicate.


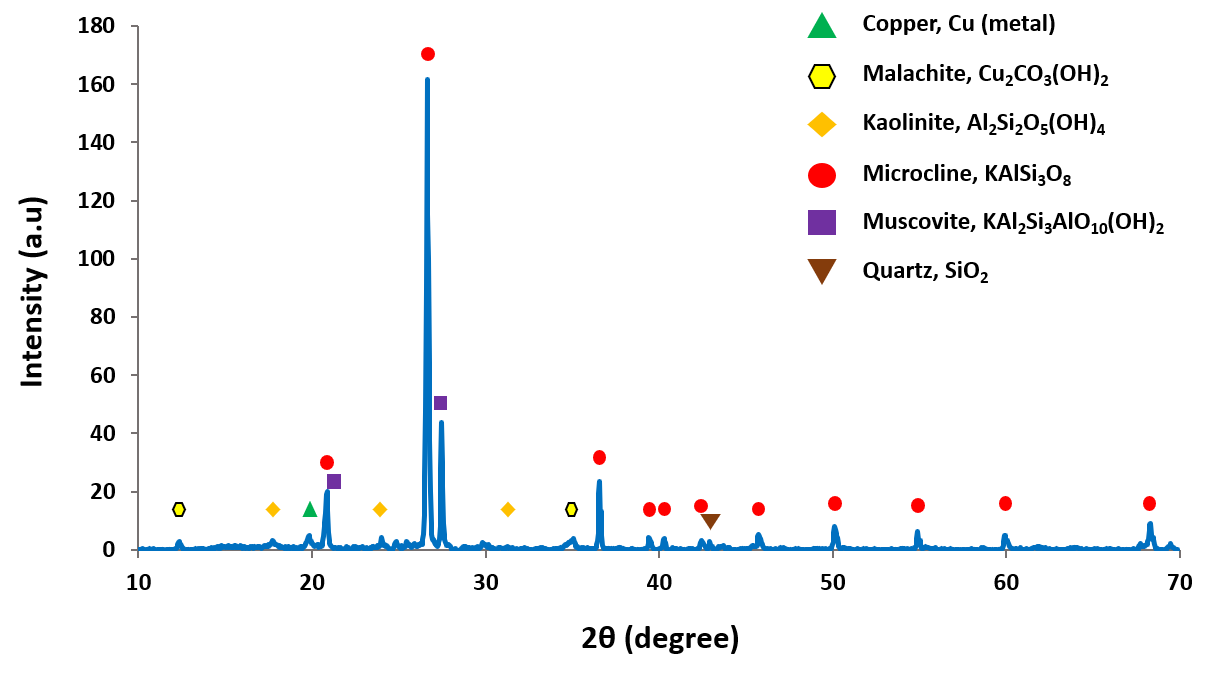


Figure S1. XRD analysis of a homogenised sample of the topsoil taken from the former mining site.


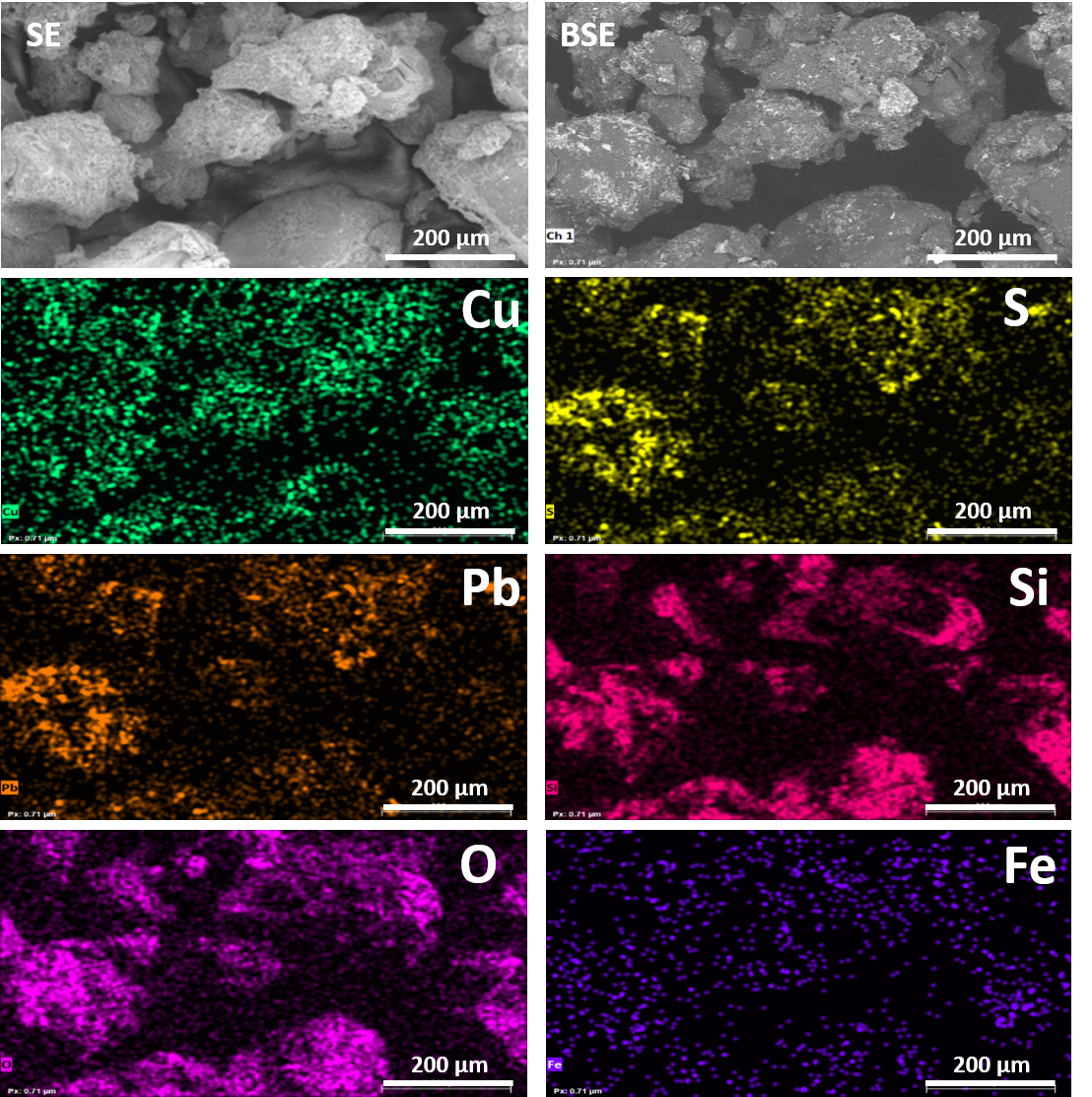


**Figure S2.** Secondary electron (SE) and backscattered electron (BSE) images of a subsample of soil taken from the topsoil of the former mining site with corresponding elemental mapping via energy-dispersive X-ray spectroscopy (EDX).


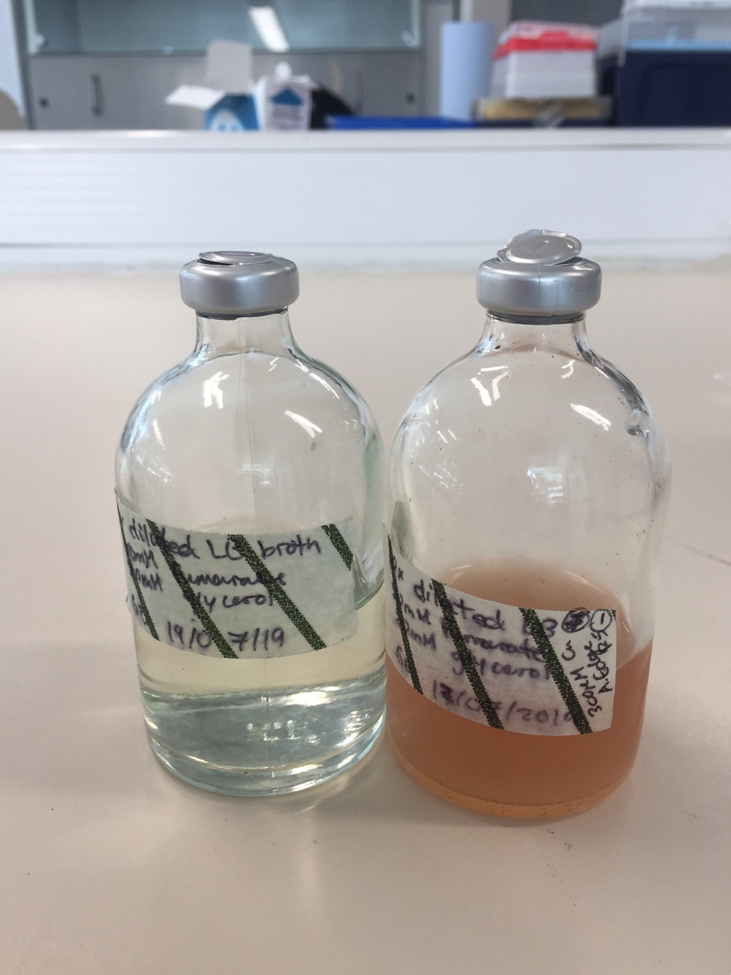


**Figure S3.** Color change observed between day zero (left) and day 7 (right) of enrichment cultures grown in a Cu-amended medium.


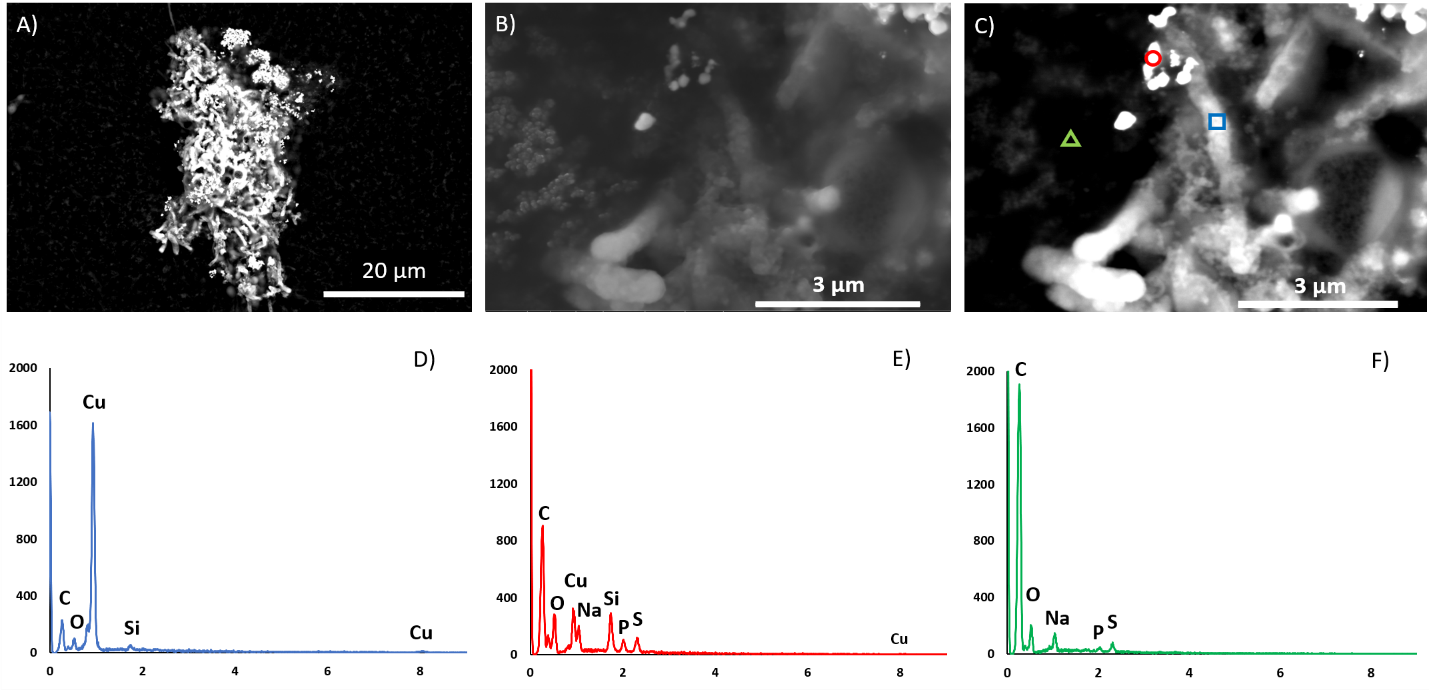


**Figure S4.** Energy-dispersive X-ray spectroscopy (EDX) point analysis of copper nanoparticles (CuNPs) acquired during environmental scanning electron microscopy (ESEM). A) Displays a cluster of cells with associated copper nanoparticles; B) and C) show a secondary electron and back-scattered image, respectively, of a magnified region in panel A. EDX point spectra (E-F) are taken from image C. D) represents the point highlighted by the blue square, E) represents the point highlighted by the red circle, and F) represents the point highlighted by the green triangle.


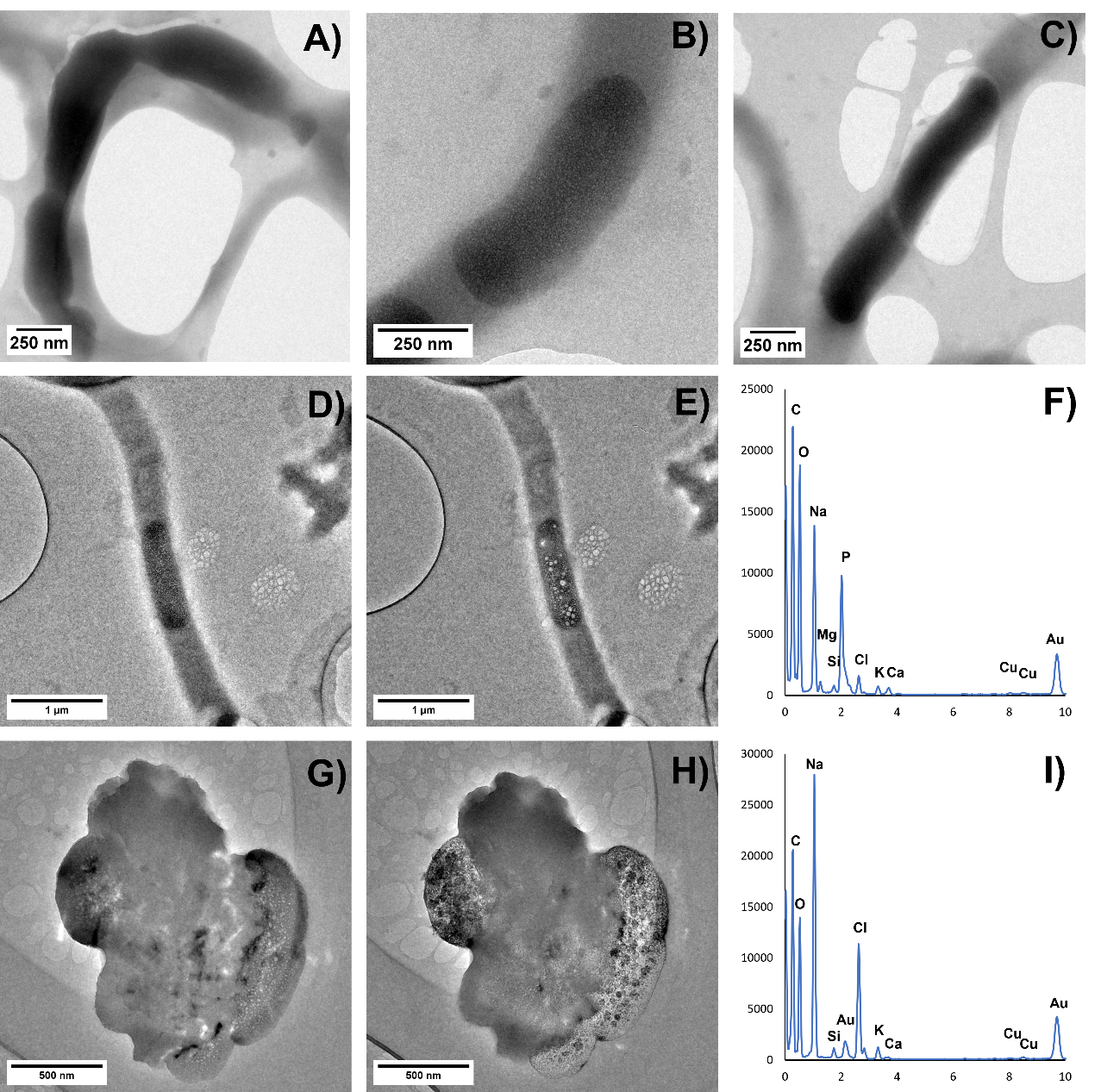


**Figure S5.** TEM images and corresponding EDX spectra acquired s from the Cu free control (A-F) and cell free control (G-I). TEM images D and G were taken prior to EDX analysis while images E and H were taken in the same location after EDX data acquisition. The Au signal is an artefact from the use of an Au mesh carbon support grid. The precipitates observed in the cell free control (G and H) are likely a result of abiotic precipitation of salts from the medium. However, no significant Cu was detected in any EDX acquisition of these precipitates (I), suggesting that Cu did not precipitate in the absence of cells. Furthermore, the high level of beam damage observed following EDX acquisition (H) suggests the precipitates are poorly crystalline in contrast to the Cu precipitates observed in the enrichment cultures.


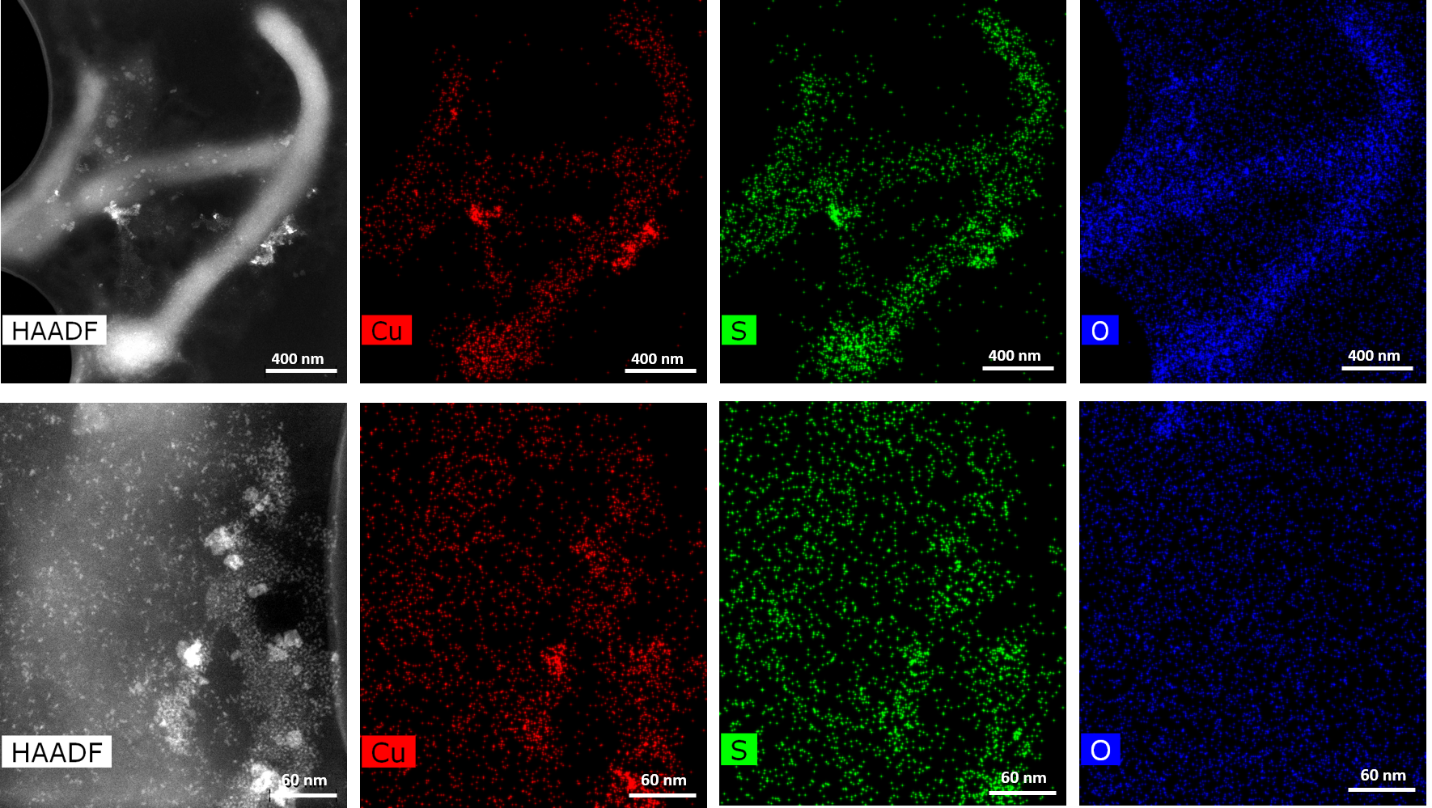


**Figure S6.** Scanning transmission electron microscope (STEM) high angle annular dark field (HAADF) images and EDX elemental maps of CuNPs associated with cells from enrichment culture 7.


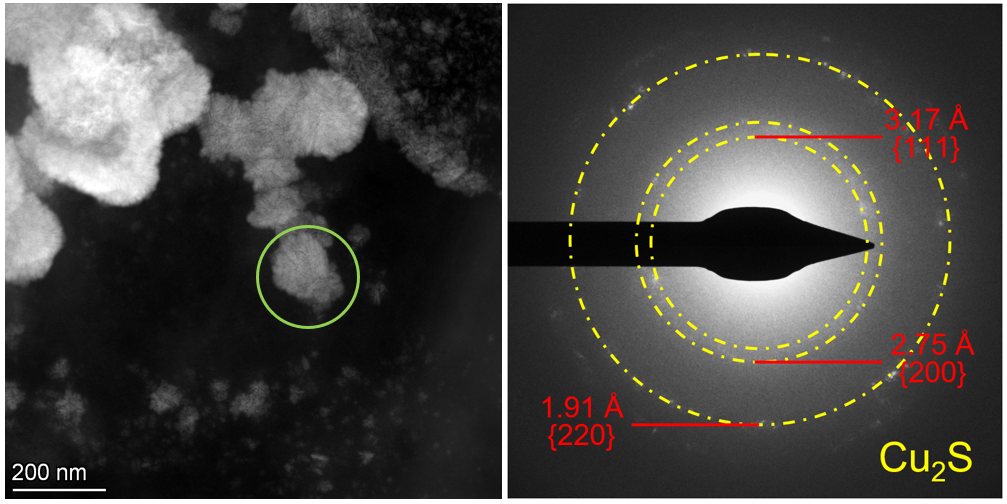


**Figure S7.** HAADF STEM image of Cu_2_S-like nanoparticles and the corresponding selected area electron diffraction pattern taken from the area marked by the green circle. The position of the observed diffraction spots corresponds to the expected Cu_2_S face centred cubic crystal structure.
